# The Wnt/β-catenin pathway regulates inflammation and apoptosis in ventilator-induced lung injury

**DOI:** 10.1042/BSR20222429

**Published:** 2023-03-09

**Authors:** Zongyu Chen, Shuang He, Siyu Lian, Yi Shen, Wenqing Jiang, Lihua Zhou, Leilei Zhou, Xianming Zhang

**Affiliations:** 1 Department of Respiratory and Critical Medicine, the Affiliated Hospital of Guizhou Medical University, Guiyang, Guizhou, China; 2 Department of Clinical Medicine, Guizhou Medical University, Guiyang, Guizhou, China; 3 Department of Respiratory and Critical Medicine, The Second People’s Hospital of Guizhou, Guiyang, Guizhou, China

**Keywords:** GSK-3β, mechanical ventilation, SB216763, ventilator-induced lung injury, Wnt/β-catenin pathway

## Abstract

Ventilator-induced lung injury (VILI) may be caused by incorrect mechanical ventilation (MV), and its progression is mainly related to inflammatory reaction, apoptosis, and oxidative stress. The Wnt/β-catenin pathway can modulate inflammation and apoptosis; however, its role in VILI is unknown. This research aims to explore the role of the Wnt/β-catenin pathway in VILI. VILI models were established using rats and type II alveolar epithelial (ATII) cells. Glycogen synthase kinase 3β (GSK-3β), β-catenin, and cyclin D1 were determined using western blotting and immunofluorescence. Apoptosis of lung tissues was evaluated using TUNEL, flow cytometry, Bax, and Bcl2 protein. Interleukin-1β (IL-1β), interleukin-6 (IL-6), and tumor necrosis factor-α (TNF-α) were detected via enzyme-linked immunosorbent assay (ELISA). Lung pathological injury was evaluated through hematoxylin and eosin (H&E) staining. Lung permeability was evaluated by the ratio of dry to wet weight of lung tissue and the total protein level of bronchoalveolar lavage fluid (BALF). The results showed that GSK-3β expression was enhanced and β-catenin expression was diminished in lung tissue under MV. SB216763 increased β-catenin and cyclin D1 expression by inhibiting GSK-3β expression and inhibited the inflammatory response and apoptosis of lung, alleviated pulmonary edema and lung tissue permeability, and significantly mitigated lung injury. However, inhibition of β-catenin expression by MSAB attenuated the anti-inflammatory and antiapoptotic effects of SB216763 in VILI. Overall, the present study demonstrates that the Wnt/β-catenin pathway activation in MV may play an anti-inflammatory and antiapoptotic role, thereby alleviating lung injury and delaying VILI progression, which may be a key point of intervention in VILI.

## Introduction

In modern medicine, invasive mechanical ventilation (MV) is an effective respiratory maintenance for critical patients [1]. However, due to the repeated action of mechanical stress, alveoli are repeatedly expanded and stretched, may cause complex changes in alveolar structure and lung tissue molecular omics, and further aggravate or induce new lung inflammation, thereby leading to ventilator-induced lung injury (VILI) [2]. Inflammatory reaction and apoptosis are important pathophysiological changes in VILI. Incorrect MV may aggravate lung tissue inflammation and apoptosis, destroy alveolar-capillary barrier and alveolar integrity, cause pulmonary tissue congestion and edema, result in lung injury, and greatly reduce the prognosis and quality of life of patients [2,3]. Despite various protective ventilation strategies are used in clinical practice [4], there is still no effective method to alleviate VILI. Thus, it is significant to explore the key pathogenesis of VILI.

The Wnt signaling pathway is a complex multibranch regulatory network, which is divided into canonical and noncanonical. The canonical pathway is mainly the Wnt/β-catenin pathway. The noncanonical pathway mainly includes the Wnt/planner cell polarity pathway and the Wnt/Ca^2+^ pathway [5]. To date, the Wnt pathway is still the focus of scientific research and is considered as an underlying regulatory target for various tumor diseases [5]. Additionally, researchers have found that it also acts an critical role on other diseases except neoplastic diseases, such as glaucoma, lung-related diseases, wound healing, skeletal diseases, etc. [6–8].

In particular, the present study pays attention to the Wnt/β-catenin pathway mediated by β-catenin. β-catenin is a multifunctional and evolutionarily conserved molecule, which is an important biomarker for judging whether this pathway is activated and is a pivotal nuclear molecule for signaling of the pathway [9]. In the cytoplasm, axis inhibitor (AXIN), glycogen synthase kinase 3β (GSK-3β), and adenomatous polyposis coli (APC) jointly promote β-catenin ubiquitination and degradation, thereby inhibiting signaling [10]. The Wnt/β-catenin pathway is critical in lung development and repair [7,11,12], and its abnormal regulation is closely correlated with lung diseases progression, such as lung cancer, bronchopulmonary dysplasia, idiopathic pulmonary fibrosis (IPF), asthma, and acute respiratory distress syndrome (ARDS) [10,13–16]. Its roles include regulating inflammation, oxidative stress, cell differentiation, and apoptosis [12,17,18]. However, no studies have elucidated whether this pathway impacts on the development of VILI.

A study has found that during the early phase of harmful tidal volume ventilation, the Wnt/β-catenin pathway was regulated in lung tissue of rats with extrapulmonary sepsis, but the specific role has not been elucidated [19]. Therefore, we hypothesized that this pathway may be correlated with the progression of VILI. In this research, the roles of this pathway in lung injury during MV were investigated in VILI models with rats and type II alveolar epithelial (ATII) cells, which may provide a strong reference for the search for VILI treatment.

## Methods and materials

### Cell experiment

Rat ATII cell line was from the Cell Bank of Chinese Academy of Sciences (Shanghai, China). High-glucose Dulbecco’s-modified Eagle’s medium (DMEM, Thermo Fisher Scientific, U.S.A.) containing 10% fetal bovine serum and 1% penicillin/streptomycin mixture provides nutrients to cells. ATII cells were cultured in an incubator at 37°C with 5% CO_2_. Experimental grouping: the control group (without cyclic stretching), high cyclic stretching with 18% mechanical stress (HCS), HCS+dimethyl sulfoxide (DMSO), HCS+DMSO+SB216763. DMSO as placebo and SB216763 as GSK-3β inhibitor (MedChemExpress, Shanghai, China). They were transfected into cells by Lipofectamine 3000 reagent (Thermo Fisher Scientific, U.S.A.) before cyclic stretching with Flexcell Tension PluFX-4000TM (Flexcell International Corporation, Burlington, U.S.A.) [20].

### Animal experiment and ethic

Thirty-six Sprague–Dawley rats (male, 6–8 weeks, weighing 240–260 g) were obtained from Beijing Hufukang Biotechnology Co., Ltd. (SCXK(Jing)2019-0008). The rats were raised with water and food in the animal room (Guizhou, China) and light/dark cycle. Then, they were grouped: the control group (preserved spontaneous breathing), low tidal volume ventilation group (LVT), high tidal volume ventilation group (HVT), HVT+DMSO, HVT+DMSO+SB216763, HVT+DMSO+SB216763+methyl 3-{[(4-methyl phenyl) sulfonyl] amino} benzoate (MSAB), MSAB as β-catenin inhibitor (MedChemExpress, Shanghai, China). There were six rats in each experimental group. Rats were intraperitoneally injected with pentobarbital (50 mg/kg, Narcoren, Merial, Germany) plus fentanyl (0.05 mg/kg, Janssen-Cilag, Neuss, Germany) to induce anesthesia; anesthesia is supplemented every hour: pentobarbital (5–10 mg/kg per hour) and fentanyl (2.5–5μg /kg per hour). After full anesthesia, the rats underwent tracheotomy for MV 4 h and were euthanized by overanesthesia. The ventilation parameters [21] were as follows: LVT was 7 ml/kg, HVT was 40 ml/kg, breathing frequency was 60 times per minute. MSAB (20 mg/kg), DMSO, and SB216763 (5 mg/kg) were injected into the abdominal cavity, respectively [22]. All animal experiments were conducted in specific pathogen free Animal Experimental Center of the Clinical Research Center of Affiliated Hospital of Guizhou Medical University. The Experimental Animal Ethics Committee of Guizhou Medical University permitted this animal experiment (number 2101324, Guizhou, China). Experiments were conducted with the National Institutes of Health’s (NIH) Guidelines for the Care and Use of Experimental Animals, and followed the Guidelines for ARRIVE.

### Lung appearance, bronchoalveolar lavage fluid, and lung wet/dry weight ratio

First, lung appearance was photographed after lung was removed. Second, using normal saline to lavaging the left lobe and collected bronchoalveolar lavage fluid (BALF), centrifuged (4°C, 1500 rpm, 10 min), and collected the supernatant to determine the total protein levels by bicinchoninic acid assay (Solarbio, Beijing, China). The right upper lobe was weighed as wet weight. Then baked it at 65°C for 72 h and weighed dry weight, and calculated W/D ratio.

### Enzyme-linked immunosorbent assay

The content of proinflammatory factor in BALF and cell culture medium was detected by enzyme-linked immunosorbent assay (ELISA) with interleukin-1β kit (IL-1β, CUSABIO, Wuhan, China), interleukin-6 kit (IL-6, CUSABIO, Wuhan, China) and tumor necrosis factor-α kit (TNF-α, CUSABIO, Wuhan, China). All procedures were follow the reagent’s instructions. Using a 450-nm spectrophotometer to determine the optical density value within 5 min.

### Hematoxylin and eosin staining

Using 4% paraformaldehyde to fix the inside and outside of the right middle lobe and stored it at 4°C for 24 h. Then used paraffin to embed the dehydrated lung tissue and sectioned it. Using hematoxylin and eosin (H&E, Solarbio, Beijing, China) to stain lung tissue sections to observe lung pathological injury by the microscope.

### TUNEL staining

The paraffin section of lung tissue was stained with a TUNEL of Apoptosis Detection Kit by the manufacturer’s instructions (Elabscicge, U.S.A.). Using the ZEN Blue system to assess the extent of apoptosis under a forward fluorescence microscope (Carl Zeiss Axio, Jena, Germany). All images were quantified by ImageJ.

### Immunofluorescence analysis

First, antigen repair was performed on lung tissue sections. Then, the tissues were washed using PBS and sealed using goat serum, and the specific primary antibodies of GSK-3β and β-catenin were instilled and deposited at 4°C for one night. Next used the fluorescent secondary antibody to incubate it in the dark and used DAPI to stain the nuclei. Fluorescence was captured under a fluorescence microscope using a ZEN blue system. All images were quantified using ImageJ software.

### Western blotting

The lower lobe and posterior lobe of lung were preserved at −80°C and analyzed by western blotting for molecular biology. Lung tissues were ground thoroughly using RIPA lysis buffer (Solarbio, Beijing, China) under an ultrasonic crusher to extract the proteins, and added loading buffer. The proteins were separated using gel electrophoresis and moved onto membranes, which was sealed with 5% skim milk for 1 h and specific primary antibodies (Proteintech, Wuhan) were added to soak membranes at 4°C for overnight, including GAPDH (1:10000), GSK-3β (1:5000), cyclin D1 (1:8000), β-catenin (1:10000), Bax (1:8000), and Bcl2 (1:1500). Then, using secondary antibody (IgG, 1:5000, Proteintech, Wuhan) to incubate the membrane and observe the imprinting via an ultrasensitive ECL chemiluminescence kit (Beyotime, P0018AM, Shanghai, China). Finally, blots were quantified by ImageJ software.

### Flow cytometry

Following the instructions, the apoptosis rate was analyzed using the Annexin V-FITC/PI Apoptosis Detection Kit (Meilunbio, Dalian, China). In a nutshell, ATII cells were digested and collected using EDTA-free trypsin and rinsed using PBS. Then, it was suspended with 1× binding buffer, added respectively Annexin V-FITC 5 μl and PI 10 μl and incubated for 15 min in the absence of light. The percentage of apoptosis was measured via flow cytometry (Beckman Navios, U.S.A.) and FlowJo software.

### Statistical analysis

Data were analyzed by GraphPad Prism 8.0.2 (GraphPad, La Jolla, CA, U.S.A.). The experiments were all repeated at least three times. Mean ± standard deviation (SD) was used to express data. Two groups were compared via Student’s *t* test, and three or more groups were compare through an ANOVA followed by a Tukey post hoc test. *P-*values<0.05 were significant.

## Results

### GSK-3β expression was increased and β-catenin expression was reduced in rat VILI models

**Figure 1:**
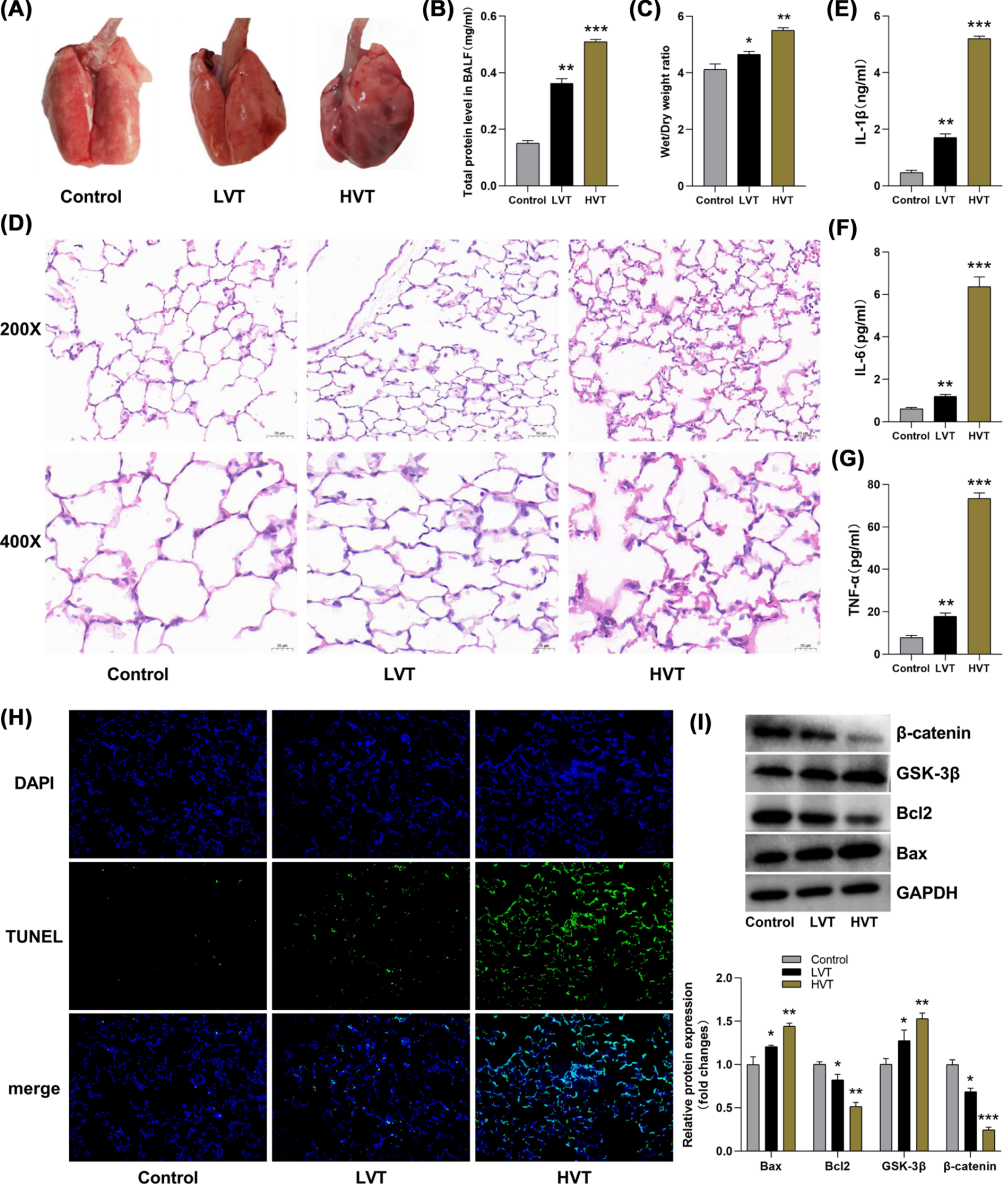
GSK-3β expression was increased and β-catenin expression was reduced in lung tissue of rats with HVT ventilation (**A**) Naked eyes directly observed lung appearance, lung was obviously congested in the HVT group. (**B,C**) Lung tissue permeability was assessed by total protein levels in BALF and lung W/D ratio (*n*=6/group). (**D**) Lung pathological injury was evaluated by H&E staining (200x and 400x). (**E–G**) The expression level of IL-1β, IL-6, and TNF-α was measured via ELISA (*n*=3/group). (**H**) Lung tissue apoptosis was examined through TUNEL staining (200x). (**I**) The expression level of Bax, Bcl2, GSK-3β, and β-catenin was examined via western blotting. GAPDH is used as an internal reference (*n*=3/group). Control vs LVT, HVT. *P<0.05, **P<0.01, ***P<0.001.

To understand whether the Wnt/β-catenin pathway is regulated in VILI, we established rat VILI models under HVT ventilation. Compared with the spontaneously breathing rats, obvious pulmonary congestion was observed after HVT ventilation, mainly manifested as lung surface was dark red color, and patchy congestion was also observed (Figure 1A). The total protein levels in BALF and lung W/D ratio were increased significantly in HVT group (Figure 1B,C). After H&E staining of lung tissue, it was observed under the microscope that the interstitial exudation of lung in the HVT group was significantly increased, with increased infiltration of inflammatory cells, alveolar rupture, and poor structural integrity of lung tissue (Figure 1D). Moreover, in the HVT group, the ELISA consequences showed that the expression of proinflammatory factor (IL-1β, IL-6, and TNF-α) in BALF were obviously raised (Figure 1E–G); TUNEL staining results indicated that the TUNEL(+)/DAPI(+) rate was obviously increased in lung tissues (Figure 1H), and the results of western blotting also indicated that Bax expression was distinctly enhanced and Bcl2 was significantly diminished (Figure 1I), lung tissue apoptosis of the HVT group was obvious. Thus, rat VILI models were successfully established in the present study. Meanwhile, western blotting results performed that GSK-3β expression was increased and β-catenin expression was reduced (Figure 1I). This indicated that this pathway is inhibited in the early stage of MV, which may be related to the development of VILI.

### Inhibition of GSK-3β could activate the Wnt/β-catenin pathway attenuates lung injury in rat VILI model

**Figure 2:**
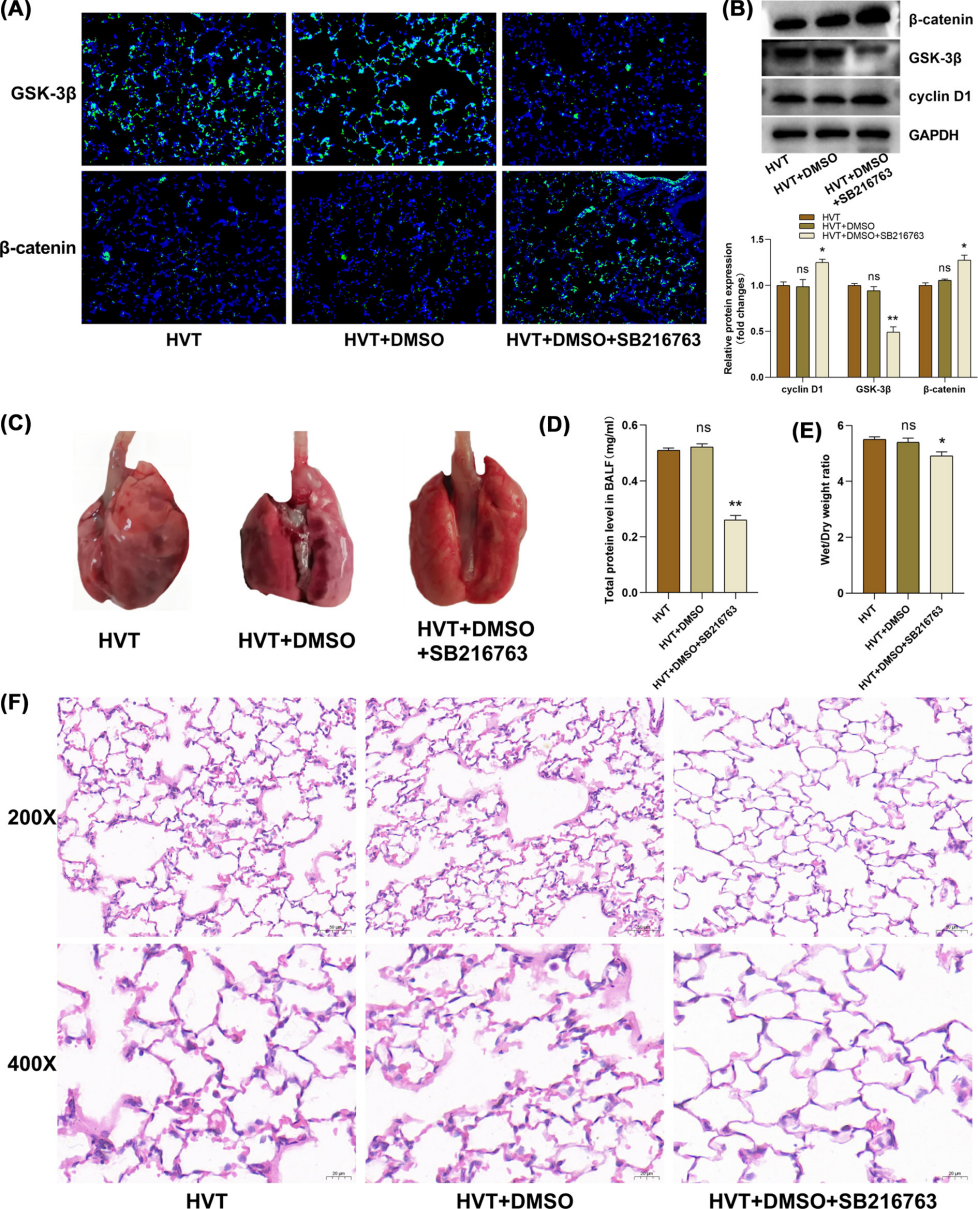
The Wnt/β-catenin pathway activation attenuated lung injury in MV. SB216763 is a GSK-3β inhibitor and DMSO as the solvent (**A,B**) The expression level of GSK-3β and β-catenin was measured through immunofluorescence (200x) and western blotting (*n*=3/group). GAPDH is used as an internal reference. (**B**) Cyclin D1 protein was detected through western blotting (*n*=3/group). (**C**) Lung appearance was visualized directly by the naked eye. (**D,E**) Lung tissue permeability was evaluated by total protein levels in BALF and lung W/D ratio (*n*=6/group). (**F**) Lung pathological injury was assessed by H&E staining (200x and 400x). HVT vs HVT+DMSO, HVT+DMSO+SB216763. ns: no statistical significance, *P<0.05, **P<0.01.

SB216763 was used to inhibit GSK-3β expression. The Wnt/β-catenin pathway-related protein expressions were measured through immunofluorescence and western blotting. Compared with the HVT group, DMSO as a solvent did not significantly affect GSK-3β, β-catenin, and cyclinD1 expression under MV (Figure 2A,B). However, GSK-3β expression was significantly inhibited with SB216763 and β-catenin and cyclin D1 expressions were significantly raised (Figure 2A,B). In addition, pulmonary congestion and bleeding were significantly mitigated by macroscopic observation of lung appearance in SB216763 group (Figure 2C), the total protein level in BALF and the W/D ratio were significantly reduced (Figure 2D,E). After H&E staining, compared with the HVT group, the lung interstitial exudation and alveolar rupture were significantly reduced in SB216763 group (Figure 2F). These results demonstrated that inhibition of GSK-3β expression by SB216763 could activate this pathway and alleviate lung injury.

### The Wnt/β-catenin pathway activation in VILI was anti-inflammatory and antiapoptotic

**Figure 3:**
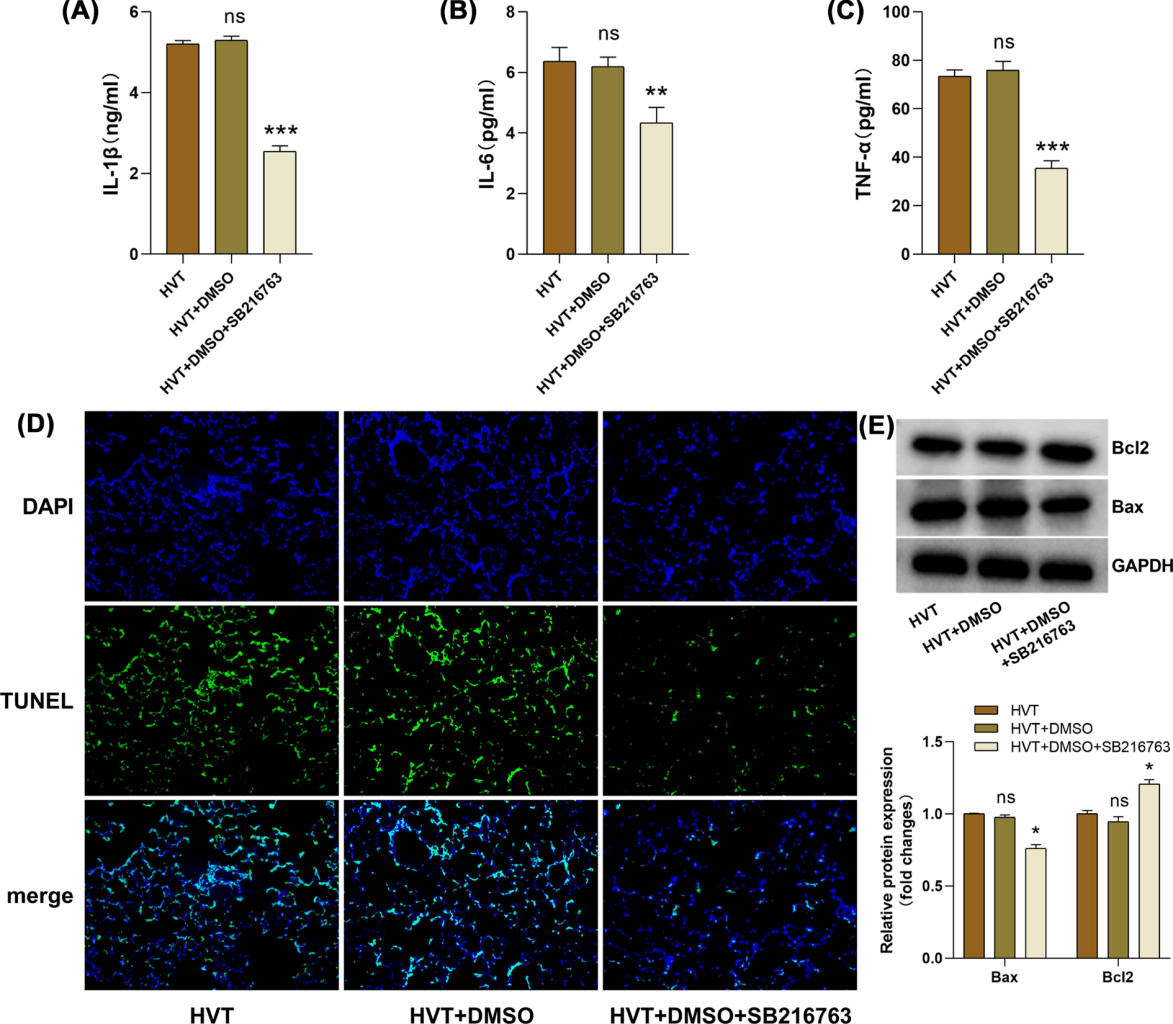
The Wnt/β-catenin pathway activation had anti-inflammatory and antiapoptotic effects in MV SB216763 is a GSK-3β inhibitor, DMSO as the solvent. (**A–C**) The expression level of IL-1β, IL-6, and TNF-α in BALF was detected through ELISA (*n*=3/group). (**D**) The degree of lung tissue apoptosis was evaluated using TUNEL staining (200x). (**E**) Bax and Bcl2 proteins were determined by western blotting, GAPDH is used as an internal reference (*n*=3/group). HVT vs HVT+DMSO, HVT+DMSO+SB216763. ns: no statistical significance, *P<0.05, **P<0.01, ***P<0.001.

Previous results have revealed that the alleviation of VILI may be correlated with the Wnt/β-catenin pathway activation, but its specific role has not been elucidated. The expression of inflammatory and apoptosis-related factors was further measured in the present study. The consequences showed that DMSO did not significantly further aggravate lung tissue inflammation and apoptosis (Figure 3A–E). Importantly, compared with the HVT group, ELISA results revealed that proinflammatory factor levels such as IL-1β, IL-6, and TNF-α were significantly reduced after SB216763 inhibited GSK-3β expression (Figure 3A–C); TUNEL staining results performed that TUNEL (+)/DAPI (+) rate was significantly reduced (Figure 3D); western blotting results demonstrated that Bax protein was obviously diminished and Bcl2 was increased (Figure 3E). The results demonstrated that the Wnt/β-catenin pathway activation under HVT ventilation plays an anti-inflammatory and antiapoptotic role, thereby alleviating lung injury and delaying VILI progression.

### Inhibition of β-catenin attenuated the anti-inflammatory and antiapoptotic effects of SB216763

**Figure 4:**
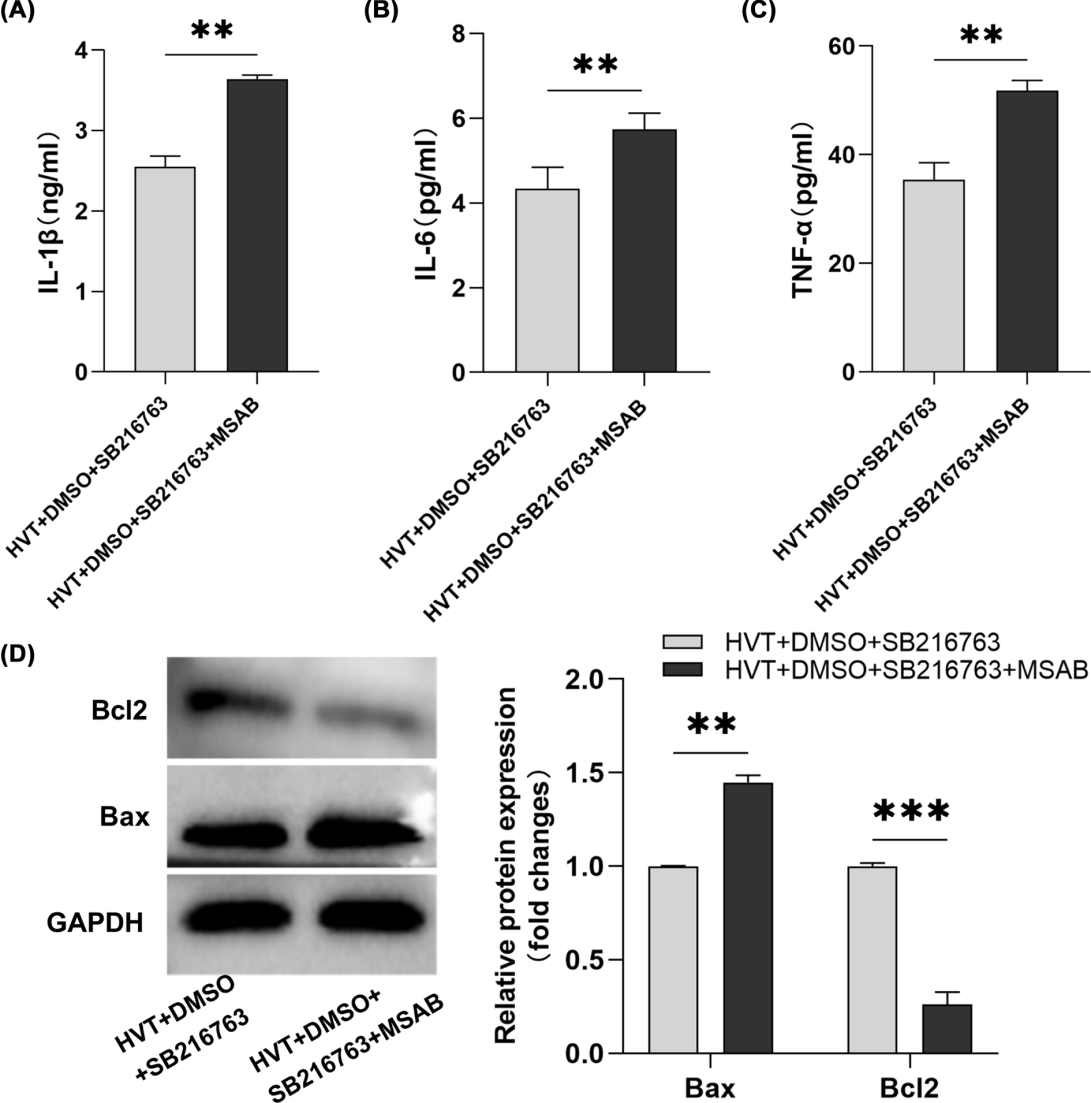
Inhibition of β-catenin attenuated the anti-inflammatory and antiapoptotic effects of SB216763 SB216763 is a GSK-3β inhibitor, MSAB is a β-catenin inhibitor, DMSO as the solvent. (**A–C**) The expression level of IL-1β, IL-6, and TNF-α in BALF was detected through ELISA (*n*=3/group). (**D**) Bax and Bcl2 proteins were determined by western blotting, GAPDH is used as an internal reference (*n*=3/group). HVT+DMSO+SB216763: GSK-3β expression inhibition group, HVT+DMSO+SB216763+MSAB: GSK-3β and β-catenin expression inhibition group. **P<0.01, ***P<0.001.

To clarify whether GSK-3β inhibitor SB216763 plays a lung protective role through β-catenin in VILI, the present study further inhibited β-catenin expression in VILI using MSAB. The results showed that compared with HVT+DMSO+SB216763 group, in HVT+DMSO+SB216763+MSAB group, the contents of IL-1β, IL-6, and TNF-α in BALF were significantly increased (Figure 4A–C), Bax expression was increased and Bcl2 expression was decreased in lung tissue (Figure 4D). In short, Figure 4 showed that after MSAB inhibited β-catenin, the anti-inflammatory and antiapoptotic effects of SB216763 in VILI were significantly weakened. Therefore, the results suggest that SB216763 plays a lung protective role at least in part by up-regulating β-catenin expression.

### The Wnt/β-catenin pathway activation was anti-inflammatory and antiapoptotic in ATII cell VILI model

**Figure 5:**
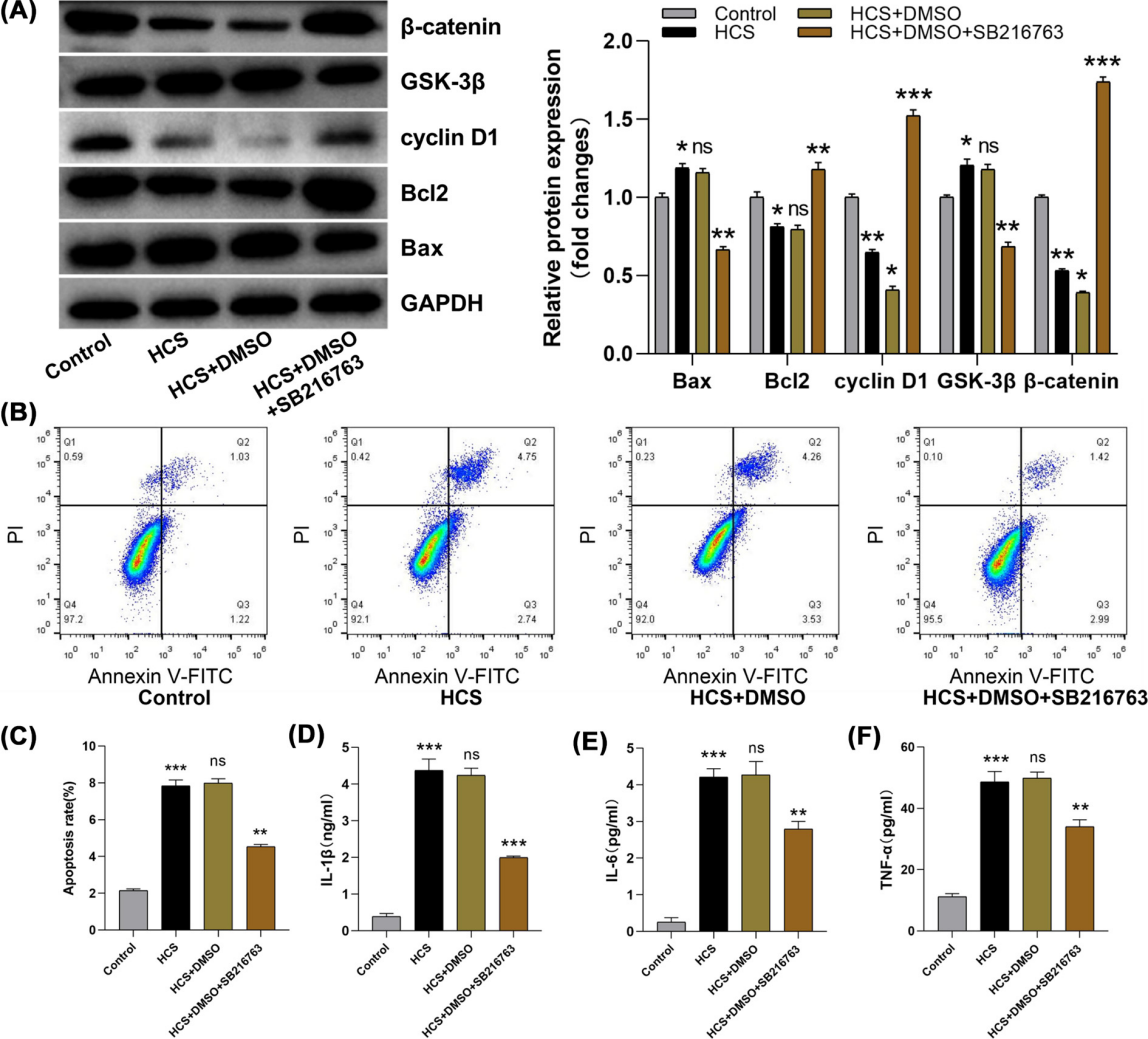
The Wnt/β-catenin pathway activation inhibited inflammation and apoptosis in ATII cell VILI model SB216763 is a GSK-3β inhibitor, DMSO as the solvent. DMSO and SB216763 were transfected into ATII cells and then cyclically stretched under 18% mechanical stress. (**A**) GSK-3β, β-catenin, cyclin D1, Bax, and Bcl2 proteins were measured via western blotting. GAPDH is used as an internal reference. (**B,C**) The apoptosis rate of ATII cell was measured through flow cytometry. (**D–F**) IL-1β, IL-6, and TNF-α was detected via ELISA. Control vs HCS, HCS vs HCS+DMSO, HCS+DMSO+SB216763. ns: no statistical significance, *P<0.05, **P<0.01, ***P<0.001.

To further clarify whether the Wnt/β-catenin pathway is correlated with VILI progression, VILI cell models were established by using ATII cells. Western blotting analysis indicated that GSK-3β protein was increased and β-catenin and cyclin D1 expression was down-regulated in ATII cells under high mechanical stress cyclic stretching compared with normal control ATII cells (Figure 5A). In addition, in ATII cells under high mechanical stress cyclic stretching, western blotting results also showed that Bax protein level was raised and Bcl2 was reduced (Figure 5A); flow cytometry analysis indicated that ATII cell apoptosis was obviously increased (Figure 5B,C); ELISA results indicated that proinflammatory factor levels were raised (Figure 5D–F). However, after transfection of SB216763 into ATII cells, GSK-3β expression was significantly inhibited and it reversed the low expression of β-catenin and cyclin D1 in the HCS group (Figure 5A). After inhibiting GSK-3β expression, western blotting results suggested that Bax was diminished and Bcl2 was increased (Figure 5A); flow cytometry results showed that ATII cell apoptosis was significantly decreased (Figure 5B,C). The expression levels of proinflammatory factor were significantly reduced (Figure 5D–F). Overall, the present study demonstrated that inhibition of GSK-3β expression could activate the Wnt/β-catenin pathway to reduce apoptosis and inflammation of ATII cells under cyclic stretching with high mechanical stress.

## Discussion

MV can maintain lung gas exchange, improve body oxygen saturation, relieve ventilator fatigue, and supply oxygen to systemic organs, which is an advanced support treatment to save the life of severe patients. However, MV can cause severe complications of VILI. Although clinically, protective ventilation strategies have been used for MV to reduce VILI, such as low tidal volume MV, high PEEP, prone position, and neuromuscular block, VILI still cannot be avoided and prevented [2]. It is a pity that there is still no effective treatment to avoid and reduce VILI, and alleviate and improve the prognosis of MV patients.

Under MV, the harmful high tidal volume causes the alveoli to be repeatedly expanded and stretched, which drives the pathophysiological processes of lung tissue such as inflammatory reaction, apoptosis, and oxidative stress, leading to the structural damage and dysfunction of lung tissue, thereby resulting in lung injury and even death of MV patients. Among them, the common harmful mediators are proinflammatory factors (IL-11β, IL-6, TNF-α) and apoptosis-related regulators (Bax, caspase-3, Bcl2). Therefore, VILI is also considered as a biological injury [2,23]. Relevant studies have shown that JAK2-STAT3, PI3/Akt, NF-κB, p38/MAPK, and other signaling pathways can mediate inflammation and/or apoptosis and act on VILI progression [20,24–27]. However, it is still necessary to explore the key points in the pathogenesis network of VILI.

The Wnt/β-catenin pathway is a intercellular signaling system and is a pivotal regulator of cell differentiation, apoptosis, and renewal, which is a key regulatory pathway for human body growth, development, repair, and maintenance of tissue homeostasis [28]. Abnormally regulated the Wnt/β-catenin pathways can aggravate or alleviate various diseases, such as glaucoma, alopecia, and fibrosis-related diseases [8,29,30]. Moreover, several researches have indicated that the Wnt/β-catenin pathway activation can regulate downstream genes to inhibit apoptosis and promote cell proliferation, thereby accelerating ovarian cancer development [31]; excessive activation of this pathway can aggravate osteoarthritis [32]; loss of this pathway during neural development can lead to diseases related to neurological dysfunction [33]. Thus, the Wnt/β-catenin pathway is regarded as a hopeful target for the traement of human diseases.

In the respiratory system, the Wnt/β-catenin pathway is a major regulator of the physiological process of lung disease, and a crucial signaling pathway throughout lung development, growth, injury, and repair, which is related to its changes or its transition to the noncanonical Wnt pathways. Stewart et al. proposed that this pathway activation can enhance resistance to chemotherapy and radiotherapy and accelerate tumor aggressiveness in non-small-cell lung cancer [13]. In a mouse model of COPD, blocking lymphotoxin β-receptor can induce regeneration through activating this pathway, thereby inhibiting apoptosis of alveolar epithelial cells [34]. Moreover, inhibiting GSK-3β expression to activate this pathway can exert an anti-inflammatory role in neonatal rats with ARDS, and promote lung repair and regeneration [16,35]. Another study has also performed that reversing the inactivation of this pathway can attenuate lipopolysaccharide (LPS)-induced apoptosis and vascular permeability of pulmonary vascular endothelial cells (EC) [36].

Given the versatility of the Wnt/β-catenin pathway, we believe that this pathway may exert a key role in VILI progression. Therefore, in the present study, animal models and cell models of VILI were built using high tidal volume and high mechanical stress, which main manifestations were pulmonary congestion and edema, increased lung permeability, significant inflammatory reaction, and apoptosis. Meanwhile, the present study performed that GSK-3β expression was increased and β-catenin was reduced in VILI, suggesting that the Wnt/β-catenin pathway was inhibited at the early phase of MV. To clarify whether its activation affects VILI progression, GSK-3β inhibitor (SB216763) was used to activate this pathway, and increase β-catenin and cyclin D1 expression. Furthermore, the Wnt/β-catenin pathway activation significantly decreased the content of IL-1β, IL-6, TNF-α, and proapoptotic protein Bax, increased Bcl2 protein, improved lung barrier function, reduced lung tissue permeability, mitigated lung congestion and edema, and significantly alleviated lung injury.

Finally, To clarify whether GSK-3β inhibitor SB216763 plays a lung protective role through β-catenin in VILI, MSAB was used to inhibit β-catenin expression. As an effective and selective inhibitor of Wnt/β-catenin pathway signaling, MSAB can bind to β-catenin and promote its degradation, thereby reducing the high expression level of active β-catenin, inhibiting its nuclear translocation, and specifically down-regulating the Wnt/β-catenin pathway target genes [37]. Our study found that inhibited β-catenin expression attenuated the anti-inflammatory and antiapoptotic effects of SB216763 in VILI. Therefore, we confirmed that SB216763 plays a lung protective role at least in part by up-regulating β-catenin expression.

However, the present study has some limitations. The molecular mechanism affecting VILI is extremely complex, in this study, it was not further elucidated whether the Wnt/β-catenin pathway interacts with JAK2-STAT3, NFκB, PI3/Akt, p38/MAPK, and other signaling pathways in VILI. In addition, whether this pathway has other roles in VILI remains to be explored. Most importantly, more attention needs to be paid to the upstream regulatory mechanisms regulating this pathway in the future.

All in all, the study shows that the Wnt/β-catenin pathway is inhibited during early MV, and activation of this pathway may exert anti-inflammatory and antiapoptotic effects, protect lung barrier function and attenuate VILI. This study may provide important reference value for VILI treatment.

## Data Availability

All data are contained within the article.

## Abbreviations

ARDS: acute respiratory distress syndrome
ATII: type II alveolar epithelial
BALF: bronchoalveolar lavage fluid
COPD: chronic obstructive pulmonary disease
GSK-3β: glycogen synthase kinase 3β
HCS: high cyclic stretching
HVT: high tidal volume
H&E: hematoxylin and eosin
IL-1β: interleukin-1β
IL-6: interleukin-6
LVT: low tidal volume
MSAB: methyl 3-{[(4-methyl phenyl) sulfonyl] amino} benzoate
MV: mechanical ventilation
TNF-α: tumor necrosis factor-α
TUNEL: TdT-mediated dUTP nick-end labeling
VILI: ventilator-induced lung injury
W/D: wet/dry weight

## References

[1] Walter K. (2021) Mechanical ventilation. JAMA 326, 1452 10.1001/jama.2021.13084 34636861

[2] Curley G.F., Laffey J.G., Zhang H. and Slutsky A.S. (2016) Biotrauma and ventilator-induced lung injury: clinical implications. Chest 150, 1109–1117 10.1016/j.chest.2016.07.019 27477213

[3] Slutsky A.S. and Ranieri V.M. (2013) Ventilator-induced lung injury. N. Engl. J. Med. 369, 2126–2136 10.1056/NEJMra1208707 24283226

[4] Brochard L., Slutsky A. and Pesenti A. (2017) Mechanical ventilation to minimize progression of lung injury in acute respiratory failure. Am. J. Respir. Crit. Care Med. 195, 438–442 10.1164/rccm.201605-1081CP 27626833

[5] Krishnamurthy N. and Kurzrock R. (2018) Targeting the Wnt/beta-catenin pathway in cancer: update on effectors and inhibitors. Cancer Treat. Rev. 62, 50–60 10.1016/j.ctrv.2017.11.002 29169144 PMC5745276

[6] Vallée A., Lecarpentier Y. and Vallée J.N. (2021) Cannabidiol and the canonical WNT/β-catenin pathway in glaucoma. Int. J. Mol. Sci. 22, 3798 10.3390/ijms22073798 33917605 PMC8038773

[7] Aros C.J., Pantoja C.J. and Gomperts B.N. (2021) Wnt signaling in lung development, regeneration, and disease progression. Commun. Biol. 4, 601 10.1038/s42003-021-02118-w 34017045 PMC8138018

[8] Huang P., Yan R., Zhang X., Wang L., Ke X. and Qu Y. (2019) Activating Wnt/β-catenin signaling pathway for disease therapy: challenges and opportunities. Pharmacol. Ther. 196, 79–90 10.1016/j.pharmthera.2018.11.008 30468742

[9] Valenta T., Hausmann G. and Basler K. (2012) The many faces and functions of β-catenin. EMBO J. 31, 2714–2736 10.1038/emboj.2012.150 22617422 PMC3380220

[10] Skronska-Wasek W., Gosens R., Königshoff M. and Baarsma H.A. (2018) WNT receptor signalling in lung physiology and pathology. Pharmacol. Ther. 187, 150–166 10.1016/j.pharmthera.2018.02.009 29458107

[11] Noack L., Bundkirchen K., Xu B., Gylstorff S., Zhou Y., Köhler K. et al. (2022) Acute intoxication with alcohol reduces trauma-induced proinflammatory response and barrier breakdown in the lung *via* the Wnt/β-catenin signaling pathway. Front. Immunol. 13, 866925 10.3389/fimmu.2022.866925 35663960 PMC9159919

[12] Zhu B., Wu Y., Huang S., Zhang R., Son Y.M., Li C. et al. (2021) Uncoupling of macrophage inflammation from self-renewal modulates host recovery from respiratory viral infection. Immunity 54, 1200.e9–1218.e9 10.1016/j.immuni.2021.04.001 33951416 PMC8192557

[13] Stewart D.J. (2014) Wnt signaling pathway in non-small cell lung cancer. J. Natl. Cancer Inst. 106, djt356 10.1093/jnci/djt356 24309006

[14] Aumiller V., Balsara N., Wilhelm J., Günther A. and Königshoff M. (2013) WNT/β-catenin signaling induces IL-1β expression by alveolar epithelial cells in pulmonary fibrosis. Am. J. Respir. Cell Mol. Biol. 49, 96–104 10.1165/rcmb.2012-0524OC 23526221

[15] Uhl F.E., Vierkotten S., Wagner D.E., Burgstaller G., Costa R., Koch I. et al. (2015) Preclinical validation and imaging of Wnt-induced repair in human 3D lung tissue cultures. Eur. Respir. J. 46, 1150–1166 10.1183/09031936.00183214 25929950

[16] Villar J., Zhang H. and Slutsky A.S. (2019) Lung repair and regeneration in ARDS: role of PECAM1 and Wnt signaling. Chest 155, 587–594 10.1016/j.chest.2018.10.022 30392791 PMC6435939

[17] Vallée A. and Lecarpentier Y. (2018) Crosstalk between peroxisome proliferator-activated receptor gamma and the canonical WNT/β-catenin pathway in chronic inflammation and oxidative stress during carcinogenesis. Front. Immunol. 9, 745 10.3389/fimmu.2018.00745 29706964 PMC5908886

[18] Reuter S., Beckert H. and Taube C. (2016) Take the Wnt out of the inflammatory sails: modulatory effects of Wnt in airway diseases. Lab. Invest. 96, 177–185 10.1038/labinvest.2015.143 26595171

[19] Villar J., Cabrera N.E., Casula M., Valladares F., Flores C., López-Aguilar J. et al. (2011) WNT/β-catenin signaling is modulated by mechanical ventilation in an experimental model of acute lung injury. Intensive Care Med. 37, 1201–1209 10.1007/s00134-011-2234-0 21567117

[20] Fan S., He J., Yang Y. and Wang D. (2022) Intermedin reduces oxidative stress and apoptosis in ventilator-induced lung injury via JAK2/STAT3. Front. Pharmacol. 12, 817874 10.3389/fphar.2021.817874 35140609 PMC8819149

[21] Chen J., Lin J., Luo H. and Li M. (2019) Effects of human interleukin-10 on ventilator-associated lung injury in rats. Inflammation 42, 538–547 10.1007/s10753-018-0911-7 30467621

[22] Zhang H., Sha J., Feng X., Hu X., Chen Y., Li B. et al. (2019) Dexmedetomidine ameliorates LPS induced acute lung injury via GSK-3β/STAT3-NF-κB signaling pathway in rats. Int. Immunopharmacol. 74, 105717 10.1016/j.intimp.2019.105717 31254953

[23] Pham T., Brochard L.J. and Slutsky A.S. (2017) Mechanical ventilation: state of the art. Mayo Clin. Proc. 92, 1382–1400 10.1016/j.mayocp.2017.05.004 28870355

[24] Wu S.W., Peng C.K., Wu S.Y., Wang Y., Yang S.S., Tang S.E. et al. (2021) Obesity attenuates ventilator-induced lung injury by modulating the STAT3-SOCS3 pathway. Front. Immunol. 12, 720844 10.3389/fimmu.2021.720844 34489970 PMC8417798

[25] Meyer N.J., Huang Y., Singleton P.A., Sammani S., Moitra J., Evenoski C.L. et al. (2009) GADD45a is a novel candidate gene in inflammatory lung injury via influences on Akt signaling. FASEB J. 23, 1325–1337 10.1096/fj.08-119073 19124556 PMC2669422

[26] Liao X., Zhang W., Dai H., Jing R., Ye M., Ge W. et al. (2021) Neutrophil-derived IL-17 promotes ventilator-induced lung injury *via* p38 MAPK/MCP-1 pathway activation. Front. Immunol. 12, 768813 10.3389/fimmu.2021.768813 34975857 PMC8714799

[27] Gaver D.P. 3rd, Nieman G.F., Gatto L.A., Cereda M., Habashi N.M. and Bates J.H.T. (2020) The POOR Get POORer: a hypothesis for the pathogenesis of ventilator-induced lung injury. Am. J. Respir. Crit. Care Med. 202, 1081–1087 10.1164/rccm.202002-0453CP 33054329 PMC7560804

[28] Steinhart Z. and Angers S. (2018) Wnt signaling in development and tissue homeostasis. Development 145, dev146589 10.1242/dev.146589 29884654

[29] Nusse R. and Clevers H. (2017) Wnt/β-catenin signaling, disease, and emerging therapeutic modalities. Cell 169, 985–999 10.1016/j.cell.2017.05.016 28575679

[30] Burgy O. and Königshoff M. (2018) The WNT signaling pathways in wound healing and fibrosis. Matrix Biol. 68-69, 67–80 10.1016/j.matbio.2018.03.017 29572156

[31] Arend R.C., Londoño-Joshi A.I., Straughn J.M. Jr and Buchsbaum D.J. (2013) The Wnt/β-catenin pathway in ovarian cancer: a review. Gynecol. Oncol. 131, 772–779 10.1016/j.ygyno.2013.09.034 24125749

[32] Stampella A., Monteagudo S. and Lories R. (2017) Wnt signaling as target for the treatment of osteoarthritis. Best Pract. Res. Clin. Rheumatol. 31, 721–729 10.1016/j.berh.2018.03.004 30509416

[33] Noelanders R. and Vleminckx K. (2017) How Wnt signaling builds the brain: bridging development and disease. Neuroscientist 23, 314–329 10.1177/1073858416667270 27624848

[34] Conlon T.M., John-Schuster G., Heide D., Pfister D., Lehmann M., Hu Y. et al. (2020) Inhibition of LTβR signalling activates WNT-induced regeneration in lung. Nature 588, 151–156 10.1038/s41586-020-2882-8 33149305 PMC7718297

[35] Li H.F. and Liu J.Y. (2019) Effects of MiR-26a on respiratory distress syndrome in neonatal rats via the wnt/β-catenin signaling pathway. Eur. Rev. Med. Pharmacol. Sci. 23, 2525–2531 30964179 10.26355/eurrev_201903_17400

[36] Lin S., Chen Q., Zhang L., Ge S., Luo Y., He W. et al. (2021) Overexpression of HOXB4 promotes protection of bone marrow mesenchymal stem cells against lipopolysaccharide-induced acute lung injury partially through the activation of Wnt/β-catenin signaling. J. Inflamm. Res. 14, 3637–3649 10.2147/JIR.S319416 34349541 PMC8326777

[37] Hwang S.Y., Deng X., Byun S., Lee C., Lee S.J., Suh H. et al. (2016) Direct targeting of β-catenin by a small molecule stimulates proteasomal degradation and suppresses oncogenic Wnt/β-catenin signaling. Cell Rep. 16, 28–36 10.1016/j.celrep.2016.05.071 27320923 PMC4957947

